# Thallusin Quantification in Marine Bacteria and Algae Cultures

**DOI:** 10.3390/md20110690

**Published:** 2022-11-01

**Authors:** Johann F. Ulrich, Melina S. Gräfe, Seema Dhiman, Paul Wienecke, Hans-Dieter Arndt, Thomas Wichard

**Affiliations:** 1 Institute of Inorganic and Analytical Chemistry, Friedrich Schiller University Jena, Lessingstr. 8, D-07743 Jena, Germany; 2 Institute of Organic Chemistry and Macromolecular Chemistry, Friedrich Schiller University Jena, Humboldtstr. 10, D-07743 Jena, Germany

**Keywords:** aquaculture, chromatography, *Maribacter*, morphogen, natural product isolation, phytohormone, seaweed, thallusin, *Ulva*, *Zobellia*

## Abstract

Thallusin, a highly biologically active, phytohormone-like and bacterial compound-inducing morphogenesis of the green tide-forming macroalga *Ulva* (Chlorophyta), was determined in bacteria and algae cultures. A sensitive and selective method was developed for quantification based on ultra-high-performance liquid chromatography coupled with electrospray ionization and a high-resolution mass spectrometer. Upon C_18_ solid phase extraction of the water samples, thallusin was derivatized with iodomethane to inhibit the formation of Fe–thallusin complexes interfering with the chromatographic separation. The concentration of thallusin was quantified during the relevant phases of the bacterial growth of *Maribacter* spp., ranging from 0.16 ± 0.01 amol cell^−1^ (at the peak of the exponential growth phase) to 0.86 ± 0.13 amol cell^−1^ (late stationary phase), indicating its accumulation in the growth medium. Finally, we directly determined the concentration of thallusin in algal culture to validate our approach for monitoring applications. Detection and quantification limits of 2.5 and 7.4 pmol L^−1^, respectively, were reached, which allow for quantifying ecologically relevant thallusin concentrations. Our approach will enable the surveying of thallusin in culture and *in nature* and will thus contribute to the chemical monitoring of aquaculture.

## 1. Introduction

The cosmopolitan green macroalga *Ulva* spp. (Ulvales, Chlorophyta) requires specifically associated bacteria that release algal growth- and morphogenesis-promoting factors (AGMPFs) [1,2]. Without such symbioses, *Ulva* develops into an undifferentiated callus under axenic conditions [1]. Because *Ulva* is commercially valuable as a source of, for example, bioactive compounds, food, and biofuel [3], beneficial algal–bacterial interactions that promote growth are of particular interest [4]. To identify such AGMPFs, a model system of *Ulva compressa* L. (cultivar *Ulva mutabilis* Føyn, recently reclassified [5]) was designed with a microbiome consisting of only two essential bacterial strains forming a tripartite community of *U. compressa*, *Roseovarius* sp. strain MS2, and *Maribacter* sp. strain MS6 [1]. *Roseovarius* sp. induces cell division in *Ulva*, while *Maribacter* sp. promotes primary rhizoid and cell wall formation. Like plant hormones, the *Roseovarius*-factor functionally resembles a cytokinin, while the *Maribacter*-factor acts auxin-like, contributing to the attachment of the algae [1,6].

Plant hormones (phytohormones) are naturally occurring small organic molecules and can be categorized into several substance classes: auxins, abscisic acid, brassinosteroids, cytokinins, ethylene, gibberellins, jasmonates, and salicylates. They are produced by plants but also by beneficial and pathogenic microorganisms (e.g., bacteria and microalgae) that can modulate plant growth, physiology, and immunity [7]. Phytohormone research is essential for advancing agricultural and marine science because many genes responsible for important agronomic traits, such as plant height, seed development, and yield, are phytohormone related [8]. 

*Maribacter* sp. releases the hormone-like compound thallusin, a terpenoid hybrid metabolite, which can functionally replace the bacterium [6]. With the half-maximal effective concentration (EC_50_) of 4.9 ± 0.1 pmol L^−1^ [9], thallusin is one of the most bioactive natural products which induces cell wall and rhizoid formation in *U. compressa* (“rhizoin”) [6,9] and thallus formation in *Gayralia oxyspermum* (“thallusin”) [10,11]. Thallusin is thus a versatile chemical regulator of plant development at low concentrations and reveals, like plant hormones [12,13], context-dependent, morphogenetic bioactivities [6,14,15] that need further investigation.

Thus, our study aimed to develop a method to determine thallusin concentration with a sufficiently low detection limit, to enable quantitative studies of *Ulva* development. A reliable quantification method for thallusin with high sensitivity and accuracy is crucial for studying the metabolic, transport, and molecular mechanisms of thallusin in marine macroalgae and, potentially, in terrestrial settings. While several modern methods exist for quantitative analysis of phytohormones in crude plant extracts that were reported [16,17,18,19], no protocols are available for thallusin. Certainly, extraction and purification protocols must be adapted for macroalgae samples [20,21,22,23]. A mass-spectrometry-based analysis is frequently influenced by the sample type (various matrices), the extraction procedure, and the experimental setup, including factors such as the chromatographic separation system and mass analyzer [24]. As such, a validated method is required to compare data generated in different laboratories and experimental setups using an appropriate internal standard.

In this contribution, we describe the development of a method for thallusin quantification in bacterial and algal growth media using an orbitrap high-resolution mass spectrometer. We studied laboratory-grown cultures of different *Maribacter* and *Zobellia* strains previously tested for thallusin production [6,25] and algal cultures. The analytical process features solid phase extraction, purification, iodomethane derivatization, and liquid chromatography–mass spectrometry (LC–MS) measurements. Notably, derivatization techniques are frequently employed in phytohormonal analysis to increase the stability and detectability of analytes [8].

## 2. Results

**Figure 1:**
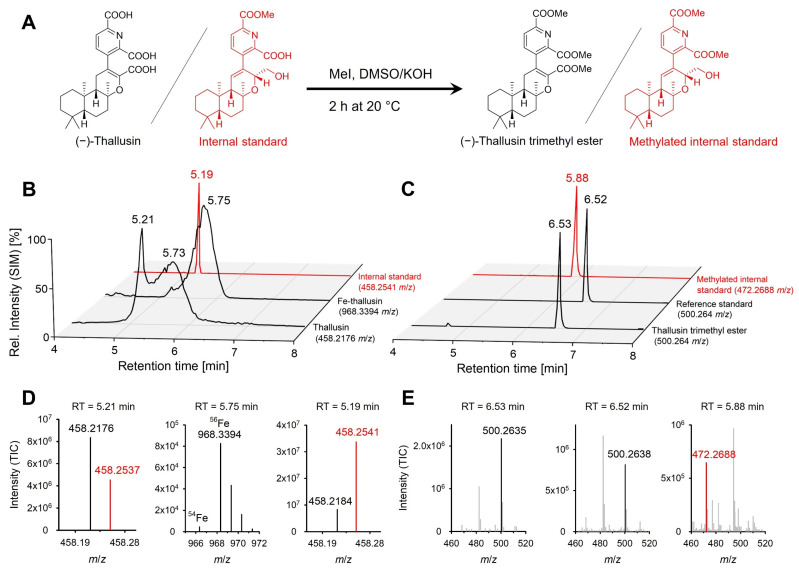
Method development for thallusin analysis and quantification by derivatization with iodomethane. (**A**) Derivatization of thallusin and the internal standard (red). (**B**) The synthetic thallusin standard (0.1 mmol L^−1^) and the internal standard (0.1 mmol L^−1^) were analyzed by UHPLC-ESI-HRMS using the single ion mode (SIM) (thallusin: *m*/*z* 458.2176; internal standard: *m*/*z* 458.2541) combined with C_18_ SPE. At 5.19 min, the internal standard was eluted, while the thallusin standard was eluted with a strong tailing after 5.21 min—the Fe–thallusin complex (*m*/*z* 968.3394) decomposed through in-source fragmentation. (**C**) Chromatograms after derivatization with iodomethane. The fully methylated thallusin trimethyl ester (*m*/*z* 500.264) and the synthetic reference standard were eluted at 6.53 min. The internal standard was eluted at 5.88 min due to methylation (*m*/*z* 472.2688). (**D**) Mass spectra (TIC) of the chromatograms are shown in (**B**). **Left**: Mass spectrum of the peak at 5.21 min—thallusin (black, *m*/*z* 458.2176) and the internal standard (red, *m*/*z* 458.2537) were detected at 5.21 min. **Middle**: ^54^Fe/^56^Fe thallusin isotope signature at 5.75 min. **Right**: Measured internal standard (red, *m*/*z* 458.2541) at 5.19 min. Thallusin (black, *m*/*z* 458.2184) was also already detectable at 5.19 min. (**E**) Mass spectra (TIC) of the chromatograms are shown in (**C**). **Left**: Mass spectrum of the peak at 6.53 min for the thallusin trimethyl ester (black, *m*/*z* 500.2635). **Middle**: Mass spectrum of the reference standard (black, *m*/*z* 500.2638) at 6.52 min. **Right**: Mass spectrum of the methylated internal standard (red, *m*/*z* 472.2688) at 5.88 min.

Thallusin and its 2:1 iron(III) complex can be detected in the bacterial culture medium and determined by LC-MS analysis, showing the characteristic pseudo molecular ions of [M + H]^+^ and [2M + Fe(III) − 2H]^+^, respectively (Figure 1) [5]. Thallusin eluted first at 5.21 min (*m*/*z* 458.2173) on a C_18_ RP silica column, directly followed by the Fe–thallusin complex (*m*/*z* 968.3388) at 5.75 min with broad tailing. Adding 0.1% (*v*/*v*) formic acid at the start of the preparation process resulted in decomplexation and improved the peak shape, but, unfortunately, it led to a 200-fold decrease in peak intensity. Notably, retention times, peak areas, and heights for metal complexes are often pH dependent; the lower the pH, the smaller the peak areas and heights. Scavenging the iron ions by using a ligand exchange reaction with ethylenediaminetetraacetic acid remained equally unsuccessful. Furthermore, thallusin and its Fe complex interfere with each other in chromatographic separations on C_18_ reversed-phase columns (Figure 1A,B). Likely, a major fraction of thallusin occurs metal bound in the environment, but its Fe(III)-complex is not kinetically stable on HPLC, as indicated by its peculiar HPLC elution profile indicating exchange. Therefore, derivatization by methylation of the carboxyl groups was investigated to suppress metal ion complexation.

### 2.1. Method Development

Methylation of the carboxyl groups of thallusin was achieved by treatment with iodomethane and potassium hydroxide in DMSO at 20 °C to give methyl esters in high yield (>99%) (Figure 1A). The thallusin trimethyl ester showed increased peak sharpness, improving the detection limit and calibration capability (Figure 1B,C). The peak was identified by comparing the associated retention time and the mass spectrum with those of a synthetic reference standard of thallusin trimethyl ester (Figure 1B–E) [9]. The added internal standard became methylated as well, thus providing evidence of successful derivatization in the analytical procedure. To estimate the methylation yield, we searched for the fully unmethylated thallusin in the total ion chromatogram (TIC). However, unmethylated thallusin and mono- (*m*/*z* 472.2330 [M + H]^+^) were not detected. The ratio of the peak areas of the double-methylated thallusin (*m*/*z* 486.2486 [M + H]^+^) to the triple (fully)-methylated thallusin (*m*/*z* 500.2643 [M + H]^+^) was 1.69 ± 0.35%; this implies that approximately 98% of the thallusin was fully methylated, close to the 99% yield obtained by Avilia-Zárraga and Martinez (2001) for derivatization of a variety of carboxylic acids by iodomethane [26].

**Figure 2:**
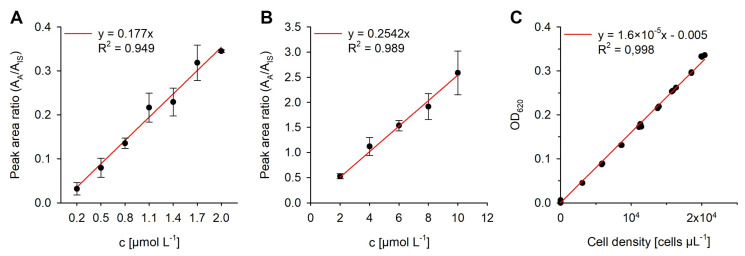
Calibration function. (**A**,**B**) Linear regression functions for the mass spectrometry data of the thallusin trimethyl ester were obtained by derivatization with iodomethane for two different working ranges. The peak area ratio of the analyte and internal standard were plotted against the concentration of the calibration standard (*n* = 3). The error bars represent the mean ± standard deviation. (**C**) For normalizing the thallusin concentration to cell number, photometric measurements of the optical density were correlated with the number of cells counted via flow cytometry.

The calibration was performed over the entire analytical process for the working range from 0.2 to 2.0 and 2.0 to 10.0 µmol L^−1^ (Figure 2A,B). The limit of detection (LOD) and limit of quantification (LOQ) were 0.0756 and 0.2217 µmol L^−1^, respectively (Table 1); these values constitute adequate detection limits to quantify thallusin in the region of the effective concentration (EC_90_ = 7.6 pmol L^−1^ [6]) in water samples. When 0.75 L of the growth medium was concentrated by solid phase extraction and the final sample volume of eluent was 25 µL, LOD and LOQ values of 2.5 and 7.4 pmol L^−1^, respectively, were obtained while accounting for a concentration factor of 30,000. The total error of the analytical procedure *s_r_* was 0.8%, indicating an accurate measurement. In order to normalize the thallusin concentration per cell, photometric optical density measurements were correlated with the number of cells counted by flow cytometry (Figure 2C). The calibrations enabled measuring thallusin concentrations in the bacterial cultures at various stages of their growth and in the laboratory-grown *Ulva* cultures.

### 2.2. Quantification of Thallusin in Bacterial Cultures

**Figure 3:**
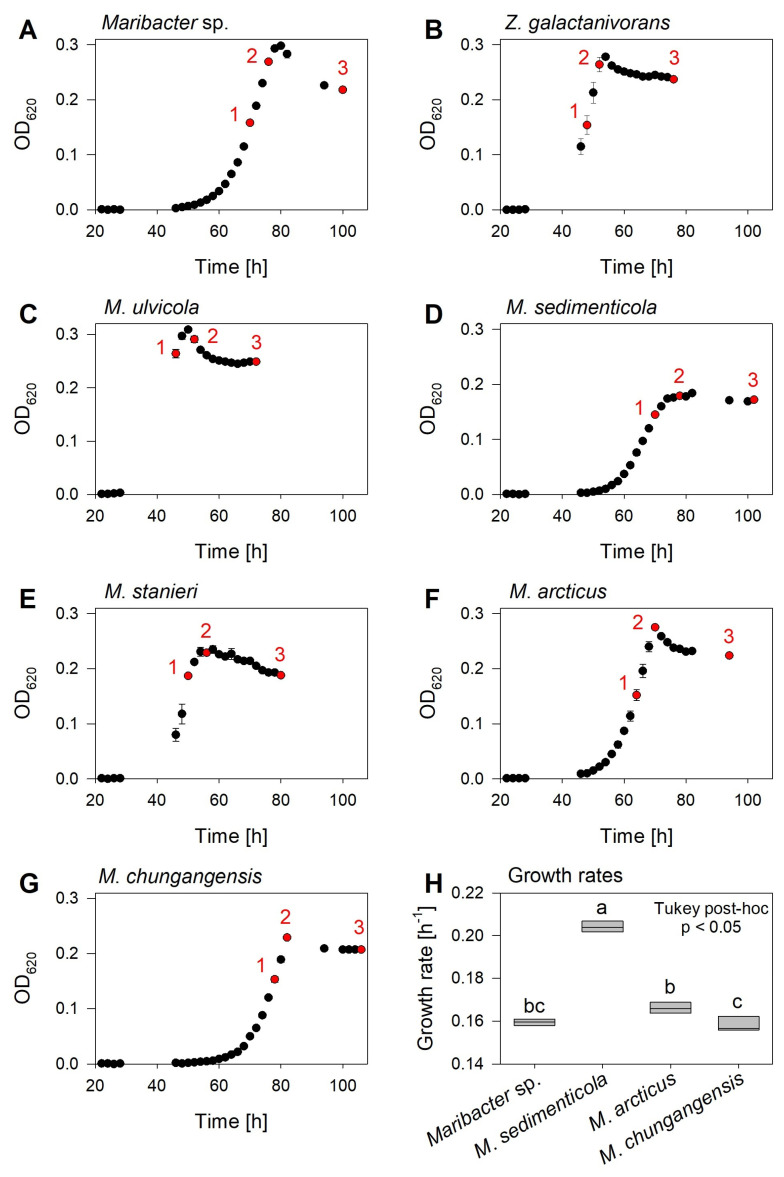
Growth curve and rates of the investigated bacterial strains. (**A**–**G**) The *Maribacter* and *Zobellia* strains were cultivated in a HaHa_100 growth medium for five days. During the different phases of growth, indicated by 1, 2, and 3, the growth medium was harvested to quantify the thallusin released (*n* = 3). (**H**) The growth rates (µ) of selected strains were determined. A one-way ANOVA determined significant differences with a Tukey post-hoc test (*p* < 0.05); the different letters on top of the boxplots indicate statistically significant differences. The error bars represent the mean ± standard deviation.

Data for the bacterial growth curves were recorded up to 24 h after reaching the stationary phase (Figure 3A–G). *Maribacter* spp. and *Zobellia* spp. were grown on the HaHa_100 medium (DSMZ 1566), which is preferred for these genera over the Marine Broth medium [21,22]. The growth of the investigated strains varied only slightly between different species. *Maribacter ulvicola* grew the fastest and reached the stationary phase after 50 h with an OD_620_ of 0.31, while *Maribacter chungangensis* grew the slowest, reaching its stationary phase after 82 h with an OD_620_ of 0.23 (growth rate μ = 0.158 ± 0.004 h^−1^). *Maribacter* sp. MS6 (max. OD_620_ = 0.30, μ = 0.160 ± 0.002 h^−1^), *Zobellia galactanivorans* (max. OD_620_ = 0.28), and *M. ulvicola* (max. OD_620_ = 0.31) yielded the highest optical densities. The growth rates (μ) between the bacteria varied significantly (one-way ANOVA, *p* < 0.05), with the highest value obtained for *Maribacter sedimenticola* (μ = 0.204 ± 0.003 h^−1^; Figure 3H).

**Figure 4:**
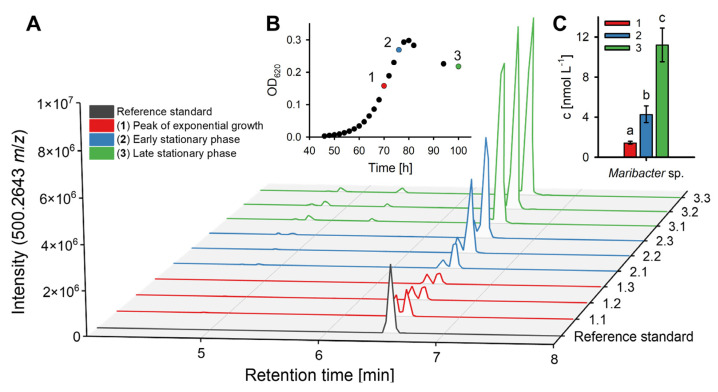
Growth-curve-derived quantification and reproducibility of biological replicates. (**A**) The UHPLC–MS chromatograms after C_18_ SPE were obtained from 50 mL of the *Maribacter* sp. MS6 supernatants, which were subsequently derivatized with iodomethane at different phases of bacterial growth (1–3). (**B**) Growth curve of *Maribacter* sp. with sampling points 1–3. (**C**) Quantification of thallusin in the culture medium during the bacterial growth phases 1–3 (*n* = 3), determined by external calibration (see also Table 2). The error bars represent the mean ± standard deviation (*n* = 3). A one-way ANOVA determined significant differences with a Tukey post-hoc test (*p* < 0.05); the different letters indicate statistically significant differences.

To quantify thallusin, samples were collected at three different growth phases. Overall, the thallusin concentration increased with the cell density and accumulated in the bacterial growth media (Figure 4).

### 2.3. Species-Dependent Production of Thallusin

**Figure 5:**
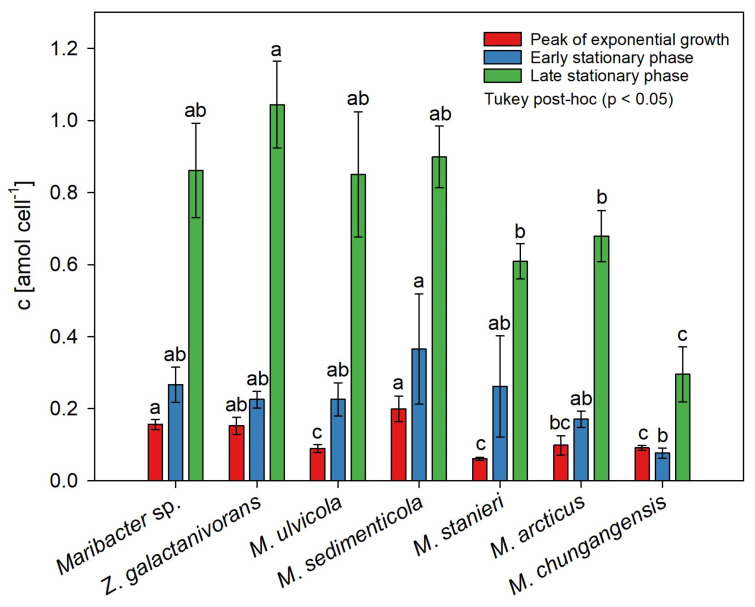
Quantification of thallusin in the growth medium normalized to the cell number for seven bacterial strains. The different letters indicate statistically significant differences in the thallusin concentration between the bacteria during a given growth phase (one-way ANOVA, Tukey post-hoc test, *p* < 0.05). The error bars represent the mean ± standard deviation (*n* = 3).

After normalization to the bacterial cell count, the thallusin concentration in the media followed a similar pattern of changes for all the tested bacteria strains, with some minor differences (compare Table 2 with Figure 5). Exemplarily, the production of thallusin in *Maribacter*’s growth medium follows the subsequent pattern: After 70 h of incubation, the supernatant of *Maribacter* sp. comprised 1.5 ± 0.1 nmol L^−1^ (0.16 ± 0.01 amol cell^−1^) thallusin at the peak of the exponential growth phase. After a further 6 h, the thallusin concentration increased significantly to 4.3 ± 0.8 nmol L^−1^ (0.27 ± 0.05 amol cell^−1^, *p* < 0.005, *t*-test, *n* = 3) before peaking at 11.2 ± 1.7 nmol L^−1^ (0.86 ± 0.13 amol cell^−1^, *p* < 0.005, *t*-test, *n* = 3) during the late stationary phase.

Overall, the supernatants of the strains *Maribacter* sp. MS6, *M. ulvicola*, *M. sedimenticola*, and *Zobellia* contained the most thallusin normalized per cell 24 h after reaching the stationary phase, with a thallusin concentration range of 9.2–14.8 nmol L^−1^ (Table 2), equivalent to approximately 0.8–1.0 amol cell^−1^ (Figure 5; one-way ANOVA, Tukey post-hoc test, *p* < 0.05). This indicates that thallusin accumulated in the medium, in agreement with previous measurements of the biological activity of *Maribacter* sp. MS6 [6,27].

### 2.4. Quantification of Thallusin in Algal Cultures

**Figure 6:**
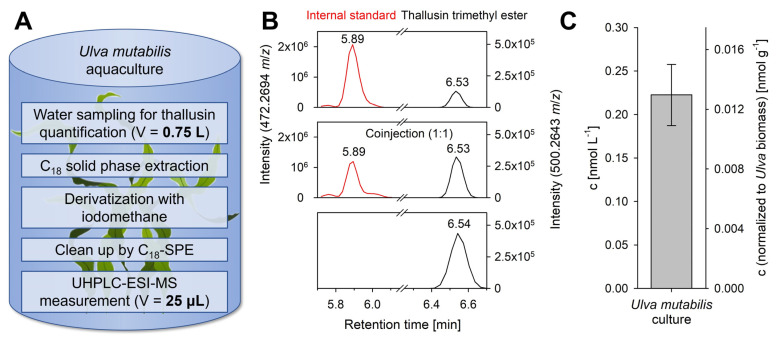
Application: Determination of thallusin in *Ulva* aquaculture. (**A**) Summary of the analytical procedure. (**B**) Algal culture: the trimethyl ester (top chromatogram) of thallusin was detected at 6.53 min by comparison with the reference standard (bottom) and by co-injection (center). (**C**) The quantification indicated a 30-fold higher concentration of thallusin than its EC_90_ value. The error bar represents the mean ± standard deviation (*n* = 3).

Using our approach, we set out to detect thallusin in the chemosphere of *Ulva* in culture. A co-injection experiment allowed for the identification of the peak of the thallusin trimethyl ester by comparison with the synthetic standard (Figure 6). The thallusin concentration in the pooled stationary culture of a closed cultivation system (*U. compressa*, three months old, 17.2 g L^−1^ fresh weight biomass) was 0.22 ± 0.04 nmol L^−1^, implying that under the selected laboratory conditions, the actual thallusin concentration in the algal culture was approximately 30-times higher than the EC_90_ = 7.6 pmol L^−1^ [9], indicating strong oversaturation.

## 3. Discussion

Methylation of thallusin was promoted by potassium hydroxide in methyl sulfoxide and iodomethane to produce the corresponding methyl ester for quantifying thallusin in bacterial and algal cultures using an orbitrap mass spectrometer (Figure 1, Table 1). The validated method was shown to be accurate with an *s_r_* < 0.8% and a sufficient LOD. As of now, 0.75 L of the medium is required to quantify thallusin levels as low as the EC_90_ value in *Ulva*’s culture (Figure 6). In fact, methylation by iodomethane has frequently been applied in analytics with good yields [28], for example, in quantifying steroidal saponins from switchgrass [29]. Other standard esterification methods use diazomethane or methanol [16], but these were not pursued due to high reagent toxicity or required harsh reaction conditions. Previously, Matsuo et al. (2005) used only trimethylsilyl diazomethane for the derivatization of thallusin preparatively [10].

Thallusin induces the development of basal rhizoids and healthy cell wall formation [6,9] in *U. compressa*, but also possesses distinguishable context-dependent functions as known for hormones in Plantae [6,30]. For plant hormone profiling, our approach should be thus included in the workflow for the qualitative and quantitative analysis of phytohormones in marine bacteria [31,32] and algae [20,33,34]. Notably, already established methods for the purification and analysis of phytohormones from culture supernatants are particularly suitable for this purpose because they are based on solid phase extractions (SPEs) starting with an Oasis^®^ HLB-sorbent SPE [35,36,37]. We confirmed that thallusin can be extracted by this sorbent as well (data not shown). However, we preferred the C_18_ SPE for thallusin analysis because it involved the extraction of fewer substances, resulting in a less structured matrix and less electrospray ion suppression than upon HLB SPE. Similarly, the bacterial growth medium HaHa_100 contributes to less background interference in the solid phase extraction, reducing ionization suppression and improving the analytical procedure and LOD. The maximum optical density of the bacterial cultures differed between the two artificial media; the growth of *Maribacter* sp. MS6 on the media led to a maximum optical density (OD_620_) of 1.78 in marine broth medium but only 0.30 in HaHa_100 (Figure 3) comparable to previously published values [38].

Quantification of thallusin in growth media will help to address whether production is dependent on amino acids and less affected by carbon and nitrogen sources, as demonstrated by the indoleacetic acid (auxin) production of epiphytic bacteria on the red alga *Pyropia yezoensis* [39]. Land-plant-associated bacteria generally produce auxin, promoting cell division in root cells, similar to thallusin [40]. Ahmed and Hasnain (2010) detected elevated concentrations of auxin—86 μg L^−1^ (0.49 mmol L^−1^)—in a *Bacillus flexus* culture shortly after the start of the stationary phase (48 h) [41], 10^7^-times higher than the thallusin concentrations at the same time point in the bacterial cultures. Similarly, Ali et al. (2008) determined auxin concentrations of 0.6 μg L^−1^ (3.4 μmol L^−1^, in *Bacillus* sp.) to 8.22 μg L^−1^ (46.9 μmol L^−1^, in *Pseudomonas* sp.) during the stationary phase by gas chromatography–mass spectrometry (GC–MS) analysis [42]; these are still 10^3^- to 10^4^-times higher than the measured concentrations of thallusin and its EC_50_ value (4.9 ± 0.1 pmol L^−1^) [9]. Therefore, the strong contrast between the high biological activity of thallusin and its comparatively low production yields is striking. For large-scale purification *Maribacter* sp., *M. ulvicola*, and *Z. galactanivorans*, the bacteria with the highest thallusin yields in this study and efficient growth performance in the HaHa_100 medium, are preferred if synthetic thallusin should be unavailable.

Because of its plant growth-promoting capabilities, the controlled use of algal biomass as biofertilizers may lessen the environmental impact of aquaculture’s excessive use of chemical fertilizers [43]. Thallusin has the potential to make a significant contribution here. The monitoring and quantification of thallusin are, thus, crucial for a deeper understanding of algae growth in complex communities and *in natura*. It should also be instrumental in studying the dynamic exchange of thallusin precursors and the active substance between algae and bacteria. As thallusin contributes to algal growth promotion, it may be used as an effective non-gene disruptive strategy for sustainable algal biorefineries [44] and a modulator of production rates of secondary metabolites [45,46].

## 4. Materials and Methods

### 4.1. Bacterial Strains and Cultivation Conditions

Bacteria isolated from *Ulva* [1] and type strains [47,48,49] (Table 2) were cultivated in a HaHa_100 medium at 20 ± 1 °C [50]. These bacteria have recently been identified as thallusin producers exhibiting typical rhizoid and cell wall formation-inducing activity [6,51].

### 4.2. Chemicals, Standards, and Materials

Methanol (MeOH, HPLC grade) and potassium hydroxide (KOH, p.a.) used for sample preparation were purchased from VWR International (Darmstadt, Germany). Dimethyl sulfoxide (DMSO) and marine broth medium were purchased from Carl Roth GmbH + Co., KG (Karlsruhe, Germany). Iodomethane was obtained from Sigma-Aldrich (Steinheim, Germany). Water (H_2_O) for the SPE was purified using a MicroPure system (J.W.T. GmbH, Jena, Germany). Thallusin and a derivative, used as internal standard, were chemically synthesized [9] and dissolved in HPLC grade MeOH to form 2.0 mmol L^−1^ stock solutions. The internal standard is a monomethylated (−)-thallusin derivative with a switched double bond and one reduced carboxylic acid (Figure 1A). The C_18_ SPE cartridges were purchased from Waters Corporation (Milford, MA, USA). All the solvents used for the sample analysis were of UHPLC grade (CHEMSOLUTE^®^) and were purchased from Th. Geyer GmbH & Co., KG (Renningen, Germany). Formic acid (≥99%) was purchased from Thermo Fisher Scientific (Rockford, IL, USA).

### 4.3. Monitoring of Bacterial Growth

The bacteria were incubated in 200 mL of a HaHa_100 medium [50,52] or marine broth [51] on an orbital shaker (230 V Euro Plug, Standard 5000, VWR). Spectrophotometric measurements were taken every 2 h at a wavelength of 620 nm using a UV-Vis spectrophotometer (Genesys 10S UV-Vis Spectrophotometer, Thermo Scientific, Waltham, MA, USA). To compute cell density, a calibration against the optical density was performed. Nine dilution steps were carried out in triplicate for *M. ulvicola* (the bacterial culture with the highest optical density) at the end of the exponential growth phase and at the peak of its optical density. The diluted bacterial samples were first analyzed with the UV-Vis spectrophotometer and directly after that with a flow cytometer (BD Accuri C6, Heidelberg, Germany) through the measurement of the forward-scattered light (50 μL, 35 μL min^−1^, threshold FSC-H 80,000) [53].

### 4.4. Sample Preparation

Samples were collected during three stages of bacterial growth to determine the thallusin concentration in the bacterial cultures. The growth medium was harvested at the peak of exponential growth (OD_620_ = 0.15), at the early (OD_620_ = 0.25) stationary phase, and a third sample was taken 24 h later during the late stationary phase. Subsequently, 50 mL aliquots of the cultures were centrifuged (9,500 rpm, 15 min, 10 °C) for sampling. The culture supernatants were then filtered sterile (pore size = 0.22 µm) and loaded onto a C_18_ reversed-phase SPE cartridge (Waters Corporation, Milford, MA, USA).

*U. compressa* L. (cultivar *Ulva mutabilis* Føyn (sl-G[mt+]; morphotype “slender”; *locus typicus*: Ria Formosa, Portugal [5]) was grown in stationary culture [25]. On average, the cultures were three months old and contained a fresh wet weight of 97.9 g of *Ulva* and 5.7 L of supernatant (17.2 g L^−1^). The culture supernatants were combined, filtered twice (using paper and cellulose nitrate filters, 0.45 µm), and subsequently divided into three 1.76 L fractions.

### 4.5. Solid Phase Extraction

The C_18_ cartridge was preconditioned with 5 mL MeOH and equilibrated with 10 mL MicroPure water. The culture supernatant was then loaded onto the cartridge. The C_18_ cartridge matrix was washed with 10 mL MicroPure water and then with 4 mL of 25% (*v*/*v*) MeOH. Subsequently, 4 mL of 75% MeOH was used allowing thallusin to elute from the matrix. The solvent of the eluates was evaporated with nitrogen (Biotage-TurboVap^®^ LV, Uppsala, Sweden), and the eluates were either directly derivatized with iodomethane or dissolved in 100 µL of 75% MeOH. The analyte solution was filtered (PVDF, 0.22 m, 4 mm, Millex^®^-GV), transferred to UHPLC-MS vials, and stored at −20 °C until measurement.

### 4.6. Derivatization of Thallusin

Thallusin was derivatized by permethylation by using iodomethane, according to the protocol of Avila-Zárraga and Martínez (2001) [26], with modifications. In brief, the dried residue of the eluates was dissolved in 150 µL of a DMSO/KOH solution (0.89 mol L^−1^), prepared by dissolving 1 g of powdered KOH (17.8 mmol) in 20 mL of DMSO with constant stirring for 30 min at 20 ± 1 °C. Iodomethane (30 µL) was then added to these sample solutions, and the mixtures were stirred for 2 h at 20 ± 1 °C. To inactivate the iodomethane, 50 µL formic acid was added to the derivatized sample, and stirring was continued for 1 h at 20 ± 1 °C. The sample mixtures were then diluted in 50 mL water and again extracted for clean-up using the C_18_ SPE protocol (see Section 4.5).

### 4.7. Ultra-High-Performance Liquid Chromatography (UHPLC) Coupled with Electrospray Ionization (ESI) High-Resolution Mass Spectrometry (HRMS) Measurements

An UltiMate HPG-3400 RS binary pump was used to perform the measurements involving UHPLC coupled with high-resolution Orbitrap MS (Thermo Fisher Scientific, Germany). The Kinetex^®^ C−18 RP chromatography column (50 × 2.1 mm; 1.7 µm; 100 Å; Phenomenex, Aschaffenburg, Germany) was kept at 25 °C within the TCC-3200 column compartment. Eluent A contained 2% acetonitrile and 0.1% (*v*/*v*) formic acid in H_2_O. Neat Acetonitrile with 0.1% (*v*/*v*) formic acid was used as eluent B. The initial conditions were as follows: 0.2 min, 0% B, flow rate of 0.4 mL min^−1^; 8.0 min, 100% B, flow rate of 0.675 mL min^−1^; 8.0–12.0 min, 100% B, flow rate of 0.675 mL min^−1^; 12.0–12.3 min, 0% B, flow rate of 0.4 mL min^−1^; and 12.3–15.0 min, 0% B, flow rate of 0.4 mL min^−1^. Under temperature-controlled conditions at 10 °C, the samples were injected using the WPS-3000 autosampler with a 25 µL injection syringe. 

Mass spectra were collected using a Thermo Fisher Scientific Q Exactive^TM^ hybrid quadrupole-Orbitrap mass spectrometer coupled to a heated ESI source. For the analysis of thallusin, the internal standard, and the methyl ester of thallusin, targeted selected ion monitoring (tSIM) was used in the positive ionization mode with the following instrument parameters: [M + H]^+^ (*m*/*z* 458.2173 ± 0.25), [M + H]^+^ (*m*/*z* 472.2694 ± 0.25), [M + H]^+^ (*m*/*z* 500.2643 ± 0.25), and [2M + Fe(III) − 2H]^+^ (*m*/*z* 968.3388 ± 0.25); resolution, 70,000; AGC target, 2 × 10^5^; maximum IT, 200 ms; and the acquisition time frame, 4.0–8.0 min. The parameters of a simultaneous full scan were set to *m*/*z* = 100–1000; resolution 70,000; AGC target, 3 × 10^6^; and maximum IT, 200 ms. Further, the sheath gas flow rate was set to 60; auxiliary gas flow rate, 20; sweep gas flow rate, 5; spray voltage, 3.0 kV; capillary temperature, 360 °C; S-lens RF level, 50; and the auxiliary gas heater temperature, 400 °C.

### 4.8. Calibration and Statistical Analysis

An external calibration curve with 5-7 calibration standards was generated in triplicate to measure the concentration of thallusin [54]. The linear regression model was calculated, plotted, and validated with SigmaPlot v.14.5 (Systat, Germany). The parameters for the calibration procedure (*s_x,o_*, *s_yx_*, *s_r_*, LOD, and LOQ) were determined according to DIN 32645. The lack-of-fit test proved linearity from the analysis of variance [55]. The test value *F* = 1.033 was smaller than the critical value *F_one-side_* = 2.958, with *P* = 95%, df*_LOF_* (lack of fit) = 5, and df*_PE_* (pure experimental error) = 14. 

## 5. Conclusions

We developed a simple, quantitative LC–HRMS method for determining the algal growth- and morphogenesis-promoting factor thallusin. Due to its high affinity to iron, the derivatization of the carboxyl groups of thallusin is essential to obtain symmetrical peaks for the analyte and efficient chromatographic separation of the analyte. The use of iodomethane for derivatization is highly effective and operationally simple. To handle matrix effects, calibration was performed throughout the entire analytical procedure. Our advanced method for the quantitative analysis of the morphogen thallusin can, thus, be used to broadly monitor thallusin in the mutualistic relationship between *Ulva* and its associated bacteria in culture as well as in natura. It may thereby contribute to a better understanding of the maintenance of sustainable aquacultures. The method is currently being expanded toward detecting thallusin in relevant bacterial and algal culture settings and should also be useful for green tide monitoring. Chemically, our study suggests that traces of other carboxylic-acid-containing metabolites released into the water may be equally quantified by implementing a similar workflow.

## Figures and Tables

**Table 1:** Selected calibration parameters for the quantification of thallusin.

Parameter	Value
Intercept, *a*_0_	0
Slope, *a*_1_	0.177
Calibration error, *s_y,x_*	0.008
Analytical standard deviation, *s_x_*_,0_	0.047 µmol L^−1^
Critical measured value, *y_c_*	0.013
Relative standard deviation, *s_r_*	0.758%
Number of calibration standards, *n_c_*	7
Limit of detection (LOD) ^1^	0.0756 µmol L^−1^
Limit of quantification (LOQ) ^1^	0.2217 µmol L^−1^

^1^ Upon processing of 0.75 L algal culture medium, the LOD and LOQ are 2.5 and 7.4 pmol L^−1^, respectively.

**Table 2:** Thallusin concentration (nmol L^−1^) in the growth media during three growth phases of various bacteria. Strain names or collection numbers are given.

			Thallusin Concentration [nmol L^−1^]		
Name	Strain	NCBI/GenBank Accession Number	OD_620_ = 0.15	OD_620_ = 0.25	Late Stationary Phase
*Maribacter* sp.	MS6	EU359911	1.46 ± 0.14	4.29 ± 0.83	11.21 ± 1.68
*Maribacter* *arcticus*	DSMZ 23546T	AY771762	0.89 ± 0.31	2.82 ± 0.37	9.08 ± 0.95
*Maribacter* *chungangensis*	CCUG 61948T	JN036550	0.82 ± 0.08	1.05 ± 0.19	3.64 ± 0.96
*Maribacter* *sedimenticola*	DSMZ 19840T	AY271623	1.71 ± 0.34	3.87 ± 1.60	9.18 ± 0.90
*Maribacter* *stanieri*	DSMZ 19891T	EU246691	0.67 ± 0.04	3.59 ± 1.98	6.79 ± 0.45
*Maribacter* *ulvicola*	DSMZ 15366T	AY271626	1.41 ± 0.15	3.96 ± 0.87	12.67 ± 2.58
*Zobellia* *galactanivorans*	DsijT	GCA_ 000973105	1.38 ± 0.22	3.57 ± 0.50	14.81 ± 1.77

## Data Availability

Not applicable.
